# *Lactobacillus plantarum* LG42 Isolated from Gajami Sik-Hae Inhibits Adipogenesis in 3T3-L1 Adipocyte

**DOI:** 10.1155/2013/460927

**Published:** 2013-02-28

**Authors:** Jeong-Eun Park, Suk-Heung Oh, Youn-Soo Cha

**Affiliations:** ^1^Department of Food Science and Human Nutrition, Chonbuk National University, Jeonju 561-756, Republic of Korea; ^2^Jeonju Makgeolli Research Center, Chonbuk National University, Jeonju 561-756, Republic of Korea; ^3^Department of Food and Biotechnology, Woosuk University, Jeonbuk 565-701, Republic of Korea

## Abstract

We investigated whether lactic acid bacteria isolated from gajami sik-hae (GLAB) are capable of reducing the intracellular lipid accumulation by downregulating the expression of adipogenesis-related genes in differentiated 3T3-L1 cells. The GLAB, *Lactobacillus plantarum* LG42, significantly decreased the intracellular triglyceride storage and the glycerol-3-phosphate dehydrogenase (GPDH) activity in a dose-dependent manner. mRNA expression of transcription factors like peroxisome proliferator-activated receptor (PPAR) **γ** and CCAAT/enhancer-binding protein (C/EBP) **α** involved in adipogenesis was markedly decreased by the GLAB treatment. Moreover, the GLAB also decreased the expression level of adipogenic markers like adipocyte fatty acid binding protein (aP2), leptin, GPDH, and fatty acid translocase (CD36) significantly. These results suggest that the GLAB inhibits lipid accumulation in the differentiated adipocyte through downregulating the expression of adipogenic transcription factors and other specific genes involved in lipid metabolism.

## 1. Introduction 

Gajami sik-hae is a fermented fish product popular in the northeastern coastal area of Korea. Sik-hae is a traditional Korean fermented seafood and has long been used for seasoning and is the generic name for a class of Korean lactic acid fermented fish products [1]. It is prepared through blending of various kinds of seafood, including cooked rice, red pepper, radish, garlic, ginger, malt meal, and salt, that becomes palatable through the subsequent preservation and fermentation [2]. The unique taste of sik-hae is due to the presence of the aforementioned ingredients and also due to the fermenting action of various microorganisms during the fermentation period [3]. Lactic acid bacteria (LAB) are the most predominant microorganisms involved in sik-hae fermentation. Some LABs were isolated from fish fermented food in Korea [4]. LAB is a viable bacteria that beneficially influence the health of the host. Recently, LAB has been revaluated for their nutritional, physiological, and pharmacological aspects, which have raised attention towards the functional effect of Korean traditional foods including kimchi and joet-gal [5]. Various nutritional and therapeutic effects ascribed to LAB in the human body are the metabolic stimulation of vitamin synthesis and enzyme production, antimutation, anticancer, gastric secretomotor, and immune function [6].

Nowadays, the consumer pays a lot of attention in looking for the relation between food and its health benefit. As a consequence, the market for foods with health-promoting properties, the so-called functional foods, has shown a remarkable growth over the past few years [7]. The development of food, which utilizes the functionality and effectiveness of probiotics, has been officially recognized as an important field of study. Consequently, numerous studies for using probiotic organisms as a functional food have been investigated. Earlier studies on probiotics have been brought into effect on the stabilization of gastrointestinal microflora, the reduction of saprogenic products, the prevention of degenerative disease, activation of the immune system, anticancer activities, anti-obesity, lowering of cholesterol, and the suppression and prevention of constipation [8–12].

*γ*-Aminobutyric acid (GABA) is a four-carbon nonprotein amino acid conserved from bacteria to plants and vertebrates. It was discovered in plants more than half a century ago, but the interest in GABA shifted to animals when it was revealed that GABA occurs at high levels in the brain, playing a major role in neurotransmission [13]. The pathway for GABA synthesis is composed of the cytosolic enzyme glutamate decarboxylase (GAD) and the mitochondrial enzymes GABA transaminase (GABA-T) and succinic semialdehyde dehydrogenase (SSADH) [14]. The consumption of GABA-enriched foods such as milk, soybean, and gabaron tea has been reported to suppress the elevation of systolic blood pressure in spontaneously hypertensive rats (SHRs) [15–17]. In this study, isolated LAB with GABA producing ability from traditional Korean fermented foods such as kimchi [18] and sik-hae (S.H. Oh). The isolated LABs were *Lactobacillus brevis* OPK-3, *Lactobacillus sakei* OPK2-59, and *Lactobacillus plantarum* LG42. Therefore, it was our interest to find whether the isolated LAB can be a novel nutraceuticals. This study was carried out with the objective of testing the antiobesity property of *Lactobacillus plantarum* LG42 (GLAB) with GABA producing ability. Our finding reveals the inhibitory effect of the GLAB on adipocyte differentiation through modulating the expression of adipogenic transcription factors and other adipogenesis specific genes, suggesting the antiobesity property of GLAB.

## 2. Materials and Methods

### 2.1. Cell Culture

 3T3-L1 cells (American Type Culture Collection (ATCC)) were cultured in DMEM containing high glucose supplemented with 10% fetal bovine serum (FBS) and penicillin/streptomycin in 6 well culture plates. Two days after confluence cells were cultured in the adipocyte differentiation cocktail media containing 5 mM 3-isobutyl-1-methylxanthine (IBMX), 1 mM dexamethasone (Sigma, USA), and insulin (10 mg/mL) in DMEM supplemented with 10% fetal bovine serum (FBS) for 2 days. The differentiation was complete after 6 days.

### 2.2. GLAB Sample and Treatment

*L. plantarum* LG42, a lactic acid bacteria having GABA producing capacity, isolated from gajami sik-hae (GLAB), was supplied by Woosuk University, genetic engineering laboratory, Korea. For TLC (thin layer chromatography) analysis, silica gel 60 F254 (Merck, Germany), standard GABA (Sigma, USA), solvent mixture (butanol: acetic acid: dichloromethanol: water in 5 : 3 : 3 : 3 ratio) were used. The cultured medium, cell-free supernatant, and cytoplasmic fraction samples (0.3 *μ*L each) were spotted 3 times. The GLAB was incubated at 37°C for 16~18 hr in MRS agar plates (Difco, Detroit, USA). All purified strains were kept at −70°C until use. After culturing the GLAB, all strains were harvested in a refrigerated centrifuge (1,100 ×g for 3 min at 4°C) and washed three times with distilled water for the removal of MRS broth. The washed GLAB was freeze-dried and resuspended in distilled water at a concentration of 10 mg/mL and homogenized for 50 sec followed by 1 min rest (repeated 3 times) using a sonicator (Fisher Scientific Co., Toronto, ON, USA). The suspension was centrifuged at 1,100  ×g for 15 min at 4°C. The 3T3-L1 cells were treated with five different concentrations of the supernatant, that is, 0 (control), 10, 20, 30, and 40 *μ*g/mL.

### 2.3. Oil Red O Staining of 3T3-L1 Adipocyte

Intracellular lipid accumulation was measured using oil red O (Sigma, St. Louis, MO). 3T3-L1 cells were fixed with 3.7% formaldehyde/PBS and stained with oil red O. Quantification of lipid accumulation was achieved by oil red O from stained cells with isopropyl alcohol and measured spectrophotometrically at 510 nm. The oil red O stained material was expressed on a per cell basis using the cell number determined from similar plates. The percentage of oil red O stained material relative to control wells containing cell culture medium without compounds was calculated as *A*_510 nm_ (GLAB)/*A*_510 nm_ (control) × 100.

### 2.4. Triglyceride Content

Triglyceride content was determined using a commercial triglyceride assay kit (Zen-bio, Research Triangle Park, NC), according to the manufacturer's protocol. The protein concentration was determined by using a Bradford reagent (Sigma, St. Louis, MO). 

### 2.5. Glycerol-3-Phosphate Dehydrogenase Activity 

GPDH was measured by following the disappearance of NADH during enzyme-catalysed dihydroxyacetone phosphate reduction using the GPDH activity assay kit (TAKARA BIO INC., Japan). GPDH activity was spectrophotometrically determined at 340 nm. One unit was defined as the amount of enzyme required for the consumption of 1 *μ*mol of NADH for one minute at 30°C. The enzyme activity was calculated with the following formula: GPDH activity (units/mL) = (ΔOD_340_ × *A* (mL) × dilution ratio of the test sample)/(6.22 × *B* (mL) × *C* (cm)).

 ΔOD_340_: decrease in the absorbance at 340 nm per minute.
 
*A* (mL): total reaction volume.
 
*B* (mL): the volume of enzyme solution (diluted sample) added.
 
*C* (cm): optical path length of the cell used*.
 6.22: millimolar absorption coefficient of NADH molecules.

### 2.6. Quantitative Real-Time PCR Analysis

Total RNA was extracted from 3T3-L1 cells at various times after adipogenic induction using Trizol reagent (Invitrogen Life Technologies; Carlsbad, CA, USA) and the concentration measured spectrophotometrically. The extracted RNA was reverse transcribed into complementary DNA using a high capacity cDNA reverse transcription kit (Applied Biosystems, Foster City, CA, USA). Then the RNA expression level was quantified by a quantitative real-time PCR using SYBR Green PCR Master Mix (Applied Biosystems, Woolston, Warrington, UK) and the 7500 real-time PCR system (Applied Biosystems, Foster City, CA, USA) according to the manufacture's protocol. The Sequences of primers used for quantitative real-time PCR are as follows: fatty acid binding protein (aP2) F: 5′-AGTGAAAACTTCGATGATTACATGAA-3′ and R: 5′-GCCTGCCACTTTCCTTGTG-3′; fatty acid translocase (CD36) F: 5′-TTGTACCTATACTGTGGCTAAATGAGA-3′ and R: 5′-CTTGTGTTTTGAACATTTCTGCTT-3′; CCAAT/enhancer-binding protein *α* (C/EBP*α*) F: 5′-AGCAACGAGTACCGGGTACG-3′ and R: 5′-TGTTTGGCTTTATCTCGGCTC-3′; peroxisome proliferator-activated receptor *γ* (PPAR*γ*) F: 5′-CAAGAATACCAAAGTGCGATCAA-3′ and R: 5′-GAGCTGGGTCTTTTCAGAATAATAAG-3′; (leptin) F: 5′-CCGCCAAGCAGAGGGTCAC-3′ and R: 5′-GCATTCAGGGCTAACATCCAACT-3′; glycerol-3-phosphate dehydrogenase(GPDH) F: 5′-CTCTTCTTGCCGCTTCAGTTT-3′ and R: 5′-CATGTAGGCCATGAGGTCCACCAC-3′; *β*-actin F: 5′-ATGGATGACGATATCGCT-3′ and R: 5′-ATGAGGTAGTCTGTCAGGT-3′. Relative quantification of gene expression with real-time PCR data was calculated relative to *β*-actin.

### 2.7. Analysis of GABA Production

GABA formation in cultured medium and cytoplasmic fraction of *L. plantarum* LG42 cells were analyzed by TLC as described (25). In order to produce GABA in the culture medium, 1% MSG was added to MRS broth and then cultured for 48 h at 30°C, and verified the existence or nonexistence of GABA in the bacterial cytoplasmic fraction by using TLC. 

### 2.8. Statistical Analysis 

All values are expressed as mean ± SD. The data were analyzed by one-way ANOVA using SPSS version 16.0. The differences among groups were assessed using Duncan's multiple range test. Statistical significance was considered at *P* value < 0.05.

## 3. Results

### 3.1. Oil Red O Staining and Intracellular Triglyceride

**Figure 1:**
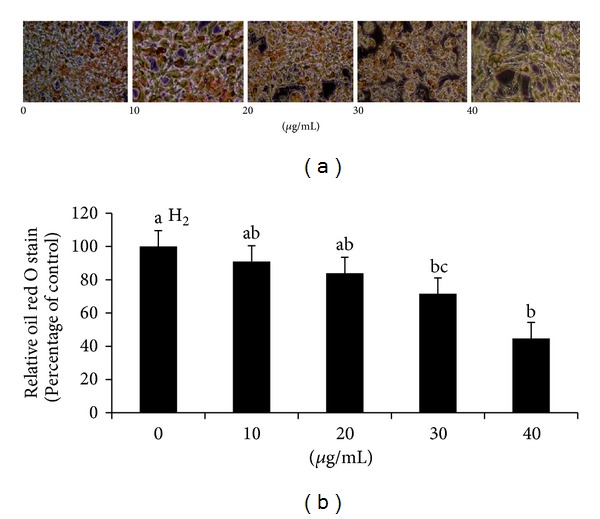
The effect of lactic acid bacteria isolated gajami sik-hae on oil red O stained in 3T3-L1 adipocyte (a) Photograph of oil red O staining. (b) Quantification of oil red O staining. Values with different superscripts are significantly different by ANOVA with Duncan's multiple range tests at *P* < 0.05.

The effects of GLAB on oil red O stained 3T3-L1 adipocyte are shown in Figure 1. Cells treated with 10 *μ*g and 20 *μ*g concentrations did not show any significant effects compared with untreated cells. Differentiated cells treated with 30 *μ*g of GLAB accumulated about a 30% decrease in intracellular lipid compared to control. Also, the treatment with 40 *μ*g GLAB resulted in further reduction to approximately 58% of the lipid accumulation of the control. 

**Figure 2:**
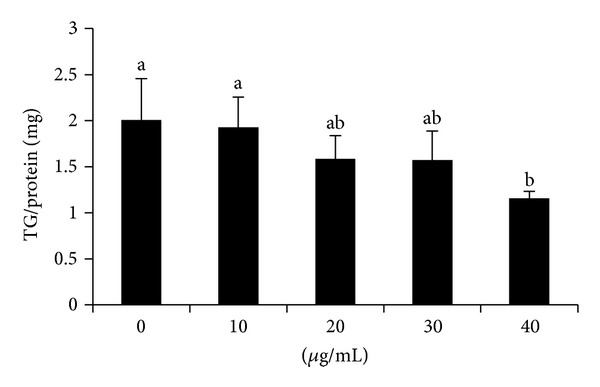
The effect of lactic acid bacteria isolated gajami sik-hae on triglyceride accumulation in 3T3-L1 adipocyte. Values with different superscripts are significantly different by ANOVA with Duncan's multiple range tests at *P* < 0.05.

The effect of GLAB on the inhibition of intracellular triglyceride in 3T3-L1 adipocyte is shown in Figure 2. The results demonstrated that GLAB caused an inhibition on intracellular triglyceride accumulation. Especially, 40 *μ*g of GLAB treatment, which was most effective compared with 10 *μ*g treated and untreated cells (Figure 2). 

### 3.2. Measurement of GPDH Activity

**Figure 3:**
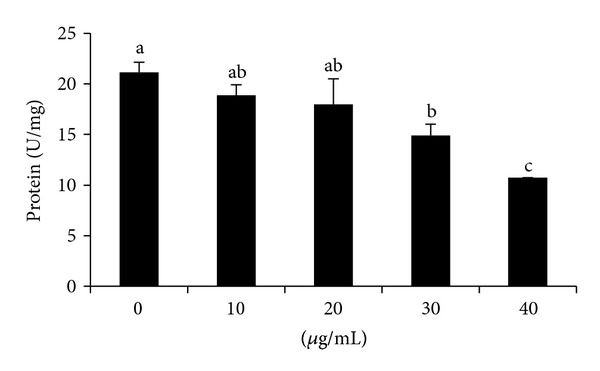
The effect of lactic acid bacteria isolated gajami sik-hae on GPDH activity in 3T3-L1 adipocyte. Values with different superscripts are significantly different by ANOVA with Duncan's multiple range tests at *P* < 0.05.

The effects of GLAB on GPDH activity in 3T3-L1 adipocyte are shown in Figure 3. GPDH activity, which indicates the late phase of adipocyte differentiation, was also determined. The effect of GLAB on adipogenesis was clearly dose dependent. The high dose, 30 and 40 *μ*g, of GLAB significantly decreased GPDH activity (Figure 3).

### 3.3. mRNA Expression of Lipid Metabolism-Related Gene in Differentiated 3T3-L1 Adipocyte

**Figure 4:**
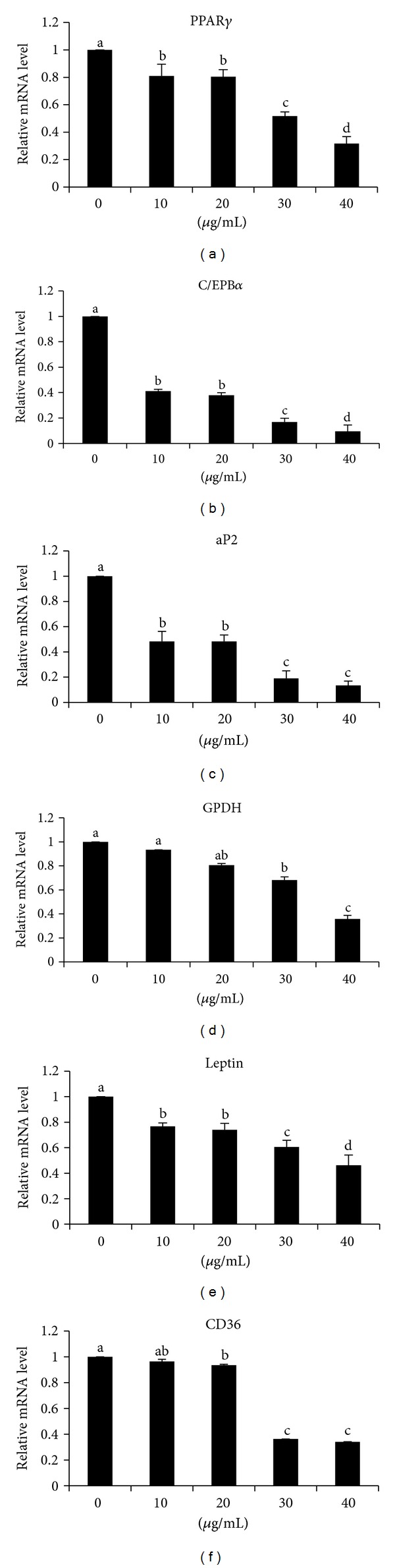
The effect of lactic acid bacteria isolated gajami sik-hae on mRNA levels of PPAR*γ*, C/EBP*α*, aP2, GPDH, leptin, and CD36 in 3T3-L1 cells. Values with different superscripts are significantly different by ANOVA with Duncan's multiple range tests at *P* < 0.05. Peroxisome proliferator-activated receptor *γ* (PPAR*γ*); CCAAT/enhancer-binding protein *α* (C/EBP*α*); fatty acid binding protein (aP2); glycerol-3-phosphate dehydrogenase(GPDH); fatty acid translocase (CD36).

PPAR*γ* and C/EBP*α* mRNA levels in differentiated 3T3-L1 adipocyte were significantly decreased in cells treated with GLAB compared with untreated cell, especially 40 *μ*g treated cell was most effective (Figure 4). In addition, the expression of PPAR*γ* target gene, aP2 was significantly decreased in GLAB treated cells, and CD 36 was significantly decreased in 20, 30, and 40 *μ*g treated cells compared with untreated cell during adipocyte differentiation (Figure 4). Cells treated with GLAB resulted in a significant decrease in the mRNA levels of leptin compared to cell untreated with GLAB. The GPDH mRNA level was significantly decreased in 30 and 40 *μ*g/mL treated cells compared with 10 *μ*g/mL treated and untreated cells.

### 3.4. GABA Production by **L. plantarum ** LG42

**Figure 5:**
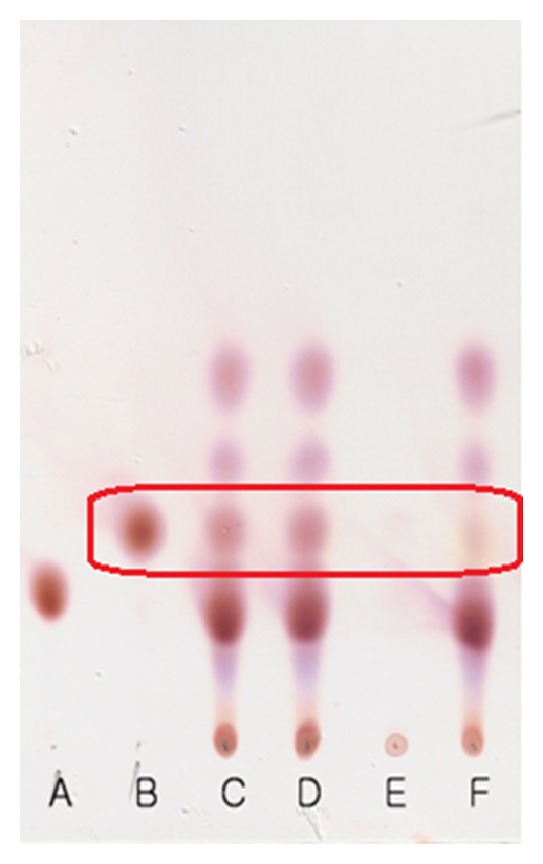
TLC analysis of GABA in the culture medium and cytoplasmic fraction of *L. plantarum* LG42 cells. A: spot of standard MSG; B: spot of standard GABA; C–E: spots of cell culture medium (C), cell-free supernatant (D), and cytoplasmic fraction of cells (E) cultured in MRS broth with 1% MSG; F: spot of cell culture medium cultured without added MSG.

GABA production by *L. plantarum* LG42 strain was studied in MRS broth containing 1% MSG. Based on the TLC analysis data, the cultured medium and cell-free culture supernatant of *L. plantarum* LG42 cells contained GABA, but not the cytoplasmic fraction (Figure 5). This data indicated that *L. plantarum* LG42 cells have GABA producing ability and the GABA was excreted mainly in the cell culture medium. 

## 4. Discussion 

Obesity threatens to become the 21st century's leading metabolic disease in the world [19]. Complications associated with obesity are responsible for the most obesity related morbidity and mortality. Obesity increases circulating cholesterol and triglyceride levels and is closely associated with hypertension, cardiovascular disease, type 2 diabetes mellitus, cancer, respiratory complications, and osteoarthritis [20]. Obesity is a condition in which adipocytes not only accumulate a large amount of fat, but also become enlarged. At a cellular level, it is characterized by an increase in the number and size of adipocytes differentiated from fibroblastic preadipocytes in the adipose tissue [21]. The severity of obesity is correlated with the degree of differentiation of preadipocytes to adipocytes and the enlargement of adipocytes in adipose tissues [22]. PPAR*γ* and C/EBP*α* are transcriptional activators, which play a major role in coordinating the adipocyte gene expression during adipocyte differentiation [23]. Therefore, we investigated the effects of lactic acid bacteria isolated gajami sik-hae (GLAB) on the differentiation of 3T3-L1 cells to clarify its antiobesity mechanism. GLAB significantly suppressed lipid accumulation in 3T3-L1 cells in a nontoxic concentration. GPDH activity was decreased in 3T3-L1 cells treated with GLAB compared to control cells incubated in the differentiation medium (Figure 3) (*P* < 0.05). GPDH is a key enzyme important for triacylglycerol synthesis [24], the present result indicates that GLAB suppresses adipocyte differentiation resulted in reducing GAPDH level. 

It is noteworthy that PPAR*γ* and C/EBP*α* levels were downregulated in 3T3-L1 cells treated with GLAB (Figure 4). PPAR*γ* and C/EBP*α* play vital roles in the early stage of adipose differentiation [25]. PPAR*γ* and C/EBP*α* regulate the expression of adipogenic genes such as CD36, leptin, GPDH, and aP2 triggering the accumulation of fat in the cells [26, 27]. In this study, the expression of PPAR*γ* and C/EBP*α* was inhibited by GLAB together resulting in reduced adipogenesis indirectly confirmed by measuring oil red stain and GPDH activity. Therefore, it appears that GLAB inhibits adipogenesis by reducing or suppressing the expression of PPAR*γ* and C/EBP*α* levels. It is also reasonable to articulate that GLAB acts directly on PPAR*γ* and C/EBP*α*. Kim et al. (2008) showed that treating 3T3-L1 cells with milk fermented with lactic acid bacteria isolated from kimchi decreased the levels of PPAR*γ* and C/EBP-*α* expression [28]. Thus, it could be possible that lactic acid bacteria isolated from gajami sik-hae affects the downstream genes of PPAR*γ* and C/EBP*α*, thus inhibiting adipogenesis in 3T3-L1 cells.

aP2 gene is central to the pathway that links obesity to insulin resistance, possibly by linking fatty acid metabolism to the expression of (tumor necrosis factor-*α*) TNF-*α* [29, 30]. CD36 mRNA expression is activated during 3T3-L1 adipocyte differentiation, and CD36 protein levels are positively correlated with PPAR*γ* and C/EBP*α* [31]. CD36 is a long chain fatty acid transporter present on the plasma membrane, as well as in intracellular pools of the skeletal muscle [32]. High level CD36 might result in lipid accumulation, which is supported by studies using CD36 null mice [33]. Leptin, a hormone and the product of the *ob* gene, is primarily secreted by adipose tissue. It is involved in the regulation of energy expenditure and food intake [34]. The expression of leptin is regulated by several substances like insulin, glucocorticoids, and TNF-*α*. Here in this study, we found that aP2, CD36, and leptin mRNA expressions were inhibited by GLAB (Figure 4). aP2, CD36, and leptin are the target genes of C/EBP*α* and PPAR*γ* and are regulated by these transcription factors. Thus, our results suggest that the inhibition of adipogenic gene expression induced by GLAB may be mediated by the inhibition of C/EBP*α* and PPAR*γ* expression.

Gajami sik-hae is a fermented food containing many kinds of microorganisms, with *Lactobacillus *and *Streptococcaceae* as the dominant species [35]. *Lactobacillus* and *Leuconostoc* species among lactobacillus produces various materials by proliferation, fermentation, and metabolism. Functional organic acids like lactic acid and citric acids are the major products of fermentation by these bacteria. Functional materials such as acetylcholine, dextran, bacteriocin, and *γ*-aminobutyric acid are also produced by these microbes depending on the origin of the fermenting materials. The fermenting materials containing carotenoids, ascorbic acids, phenolic compounds, and amino acids play a major role in this process [18, 36]. Among the various beneficial health effects of the probiotics [37, 38], their biological impact on obesity has generated a considerable interest. Regarding the anti-obesity property of probiotics, some reports demonstrated that dairy products fermented with lactic acid bacteria exert anti-obesity effects [28, 39, 40]. Previously, it has been shown that cytoplasmic fractions of useful LAB have some beneficial effects for the improvement of health related symptoms [41, 42]. Among various compounds from LAB contributing to anti-obesity effects and improving lipid profiles are conjugated linoleic acid (CLA), ornithine, GABA, hydroxy methyl glutaric acid, orotic acid, and so forth [18, 43–46]. Although GABA can exert an anti-obesity effect and the GLAB has GABA producing capacity and since the cytoplasmic fraction used in this study contains a negligible amount of GABA (Figure 5), it is not clear what compounds of GLAB worked as the main principles of the anti-obesity effects. Therefore, GLAB having GABA producing ability can be used as a useful material not only for the production of fermented foods such as sik-hae with an enhanced level of GABA, but also for the investigation of unknown compounds except GABA for the anti-obesity effects. Further studies are required to identify the active compounds of GLAB that have specific effects on obesity. 

In conclusion, the inhibitory effects of GLAB on 3T3-L1 adipocyte, as indicated by a decrease in intracellular triglyceride content and GPDH activity have been elucidated. It appears to be mediated through downregulating the expression of adipogenic transcription factor, PPAR*γ* and C/EBP*α*, and adipocyte-specific gene such as aP2, leptin, GPDH, and CD36. These results indicate that GLAB may play a role in the control of adipogenesis and might have further implication for *in vivo* antiobesity effect.
